# Prosthetic Retention Mode and Peri-Implant Marginal Bone Level at a Minimum Five-Year Follow-Up After Maxillary Sinus Floor Elevation: A Retrospective Clustered Cohort Study

**DOI:** 10.3390/jfb17090463

**Published:** 2026-09-09

**Authors:** Cristian Niky Cumpătă, Călin Rareș Roman, Mihaela Jana Țuculină, Ioana Smaranda Cumpătă, Alexandru Burcea, Cristina Maria Munteanu, Mădălina Anca Moldovan, Adrian Camen, Sebastian Petrescu, Paolo Di Francesco

**Affiliations:** 1 Department of Oral and Maxillofacial Surgery, Faculty of Dental Medicine, Titu Maiorescu University, 031593 Bucharest, Romania; nikycumpata@yahoo.com; 2 Department of Oro-Maxillo-Facial Surgery and Implantology, Faculty of Dental Medicine, Iuliu Hațieganu University, 400012 Cluj-Napoca, Romania; raresroman25@gmail.com (C.R.R.); madalina.lazar@umfcluj.ro (M.A.M.); 3 Department of Endodontics, Faculty of Dental Medicine, University of Medicine and Pharmacy of Craiova, 200349 Craiova, Romania; mtuculina@yahoo.com; 4 Faculty of Dental Medicine, Titu Maiorescu University, 031593 Bucharest, Romania; smarandacumpata@yahoo.com; 5 Department of Speciality Disciplines, Faculty of Dental Medicine, Titu Maiorescu University, 031593 Bucharest, Romania; alexandru.burcea@prof.utm.ro; 6 Department of Oro-Maxillo-Facial Surgery and Implantology, Faculty of Dental Medicine, University of Medicine and Pharmacy of Craiova, 200349 Craiova, Romania; cristina.munteanu@umfcv.ro (C.M.M.); adrian.camen@umfcv.ro (A.C.); sebastian.petrescu@umfcv.ro (S.P.); 7 Department of Prosthodontics, Faculty of Dental Medicine, Titu Maiorescu University, 031593 Bucharest, Romania; dr.difrancescopaolo@gmail.com

**Keywords:** dental implant, cement-retained restoration, screw-retained restoration, prosthetic retention, marginal bone level, maxillary sinus floor elevation, generalized Festimating equations, retrospective cohort

## Abstract

**Background/Objectives:** Evidence comparing cement- and screw-retained implant-supported restorations remains heterogeneous, particularly in augmented posterior maxillae. This study evaluated the association between definitive retention mode and peri-implant marginal bone level at a minimum 5-year follow-up after maxillary sinus floor elevation. **Methods:** This retrospective clustered cohort included 248 patients and 528 bone-level implants; radiographic measurements were available for 522 implants from 247 patients. Marginal bone level was assessed by CBCT immediately after implant placement and at least 60 months later. The primary adjusted analysis used generalized estimating equations with patient-level clustering and robust standard errors. **Results:** Mean marginal bone level at final follow-up was 0.133 mm for cement-retained and 0.105 mm for screw-retained restorations; the unadjusted difference was not statistically significant (Mann–Whitney U = 31,366; *p* = 0.064). In the primary GEE model, which excluded provisional retention because of its strong collinearity with definitive retention, definitive screw retention was not associated with marginal bone level at follow-up (B = −0.0028 mm; 95% CI: −0.0165–0.0108; *p* = 0.684). The time × definitive retention interaction was also not significant (B = −0.0069 mm; 95% CI: −0.0187–0.0049; *p* = 0.249). **Conclusions:** Definitive retention mode was not significantly associated with marginal bone level in the primary adjusted analysis, and longitudinal marginal bone change did not differ significantly between retention strategies. Sensitivity analyses indicated that estimates were influenced by the strong collinearity between provisional and definitive retention. These observational findings do not demonstrate superiority or equivalence of either retention strategy.

## 1. Introduction

Fixed implant-supported restorations can be retained by cementation to prosthetic abutments or by direct screw retention. Both strategies are well established and can provide favorable clinical outcomes, but they differ in their biological and technical characteristics. Screw retention facilitates retrievability, component control, and maintenance procedures, whereas cement retention may facilitate occlusal design without a screw-access channel and may be advantageous when implant angulation does not allow favorable positioning of the access opening [1,2].

Evidence comparing the two retention strategies has not established a consistent difference in peri-implant marginal bone outcomes. Systematic reviews and meta-analyses have reported either small differences between cement- and screw-retained restorations or no clinically relevant difference, with interpretation limited by heterogeneity in restoration type, implant position, restorative materials, and follow-up duration [2,3,4]. Retention mode should therefore be considered within the broader prosthetic and peri-implant context rather than as an isolated determinant of marginal bone behavior.

The principal biological concern associated with cement-retained restorations is the persistence of excess cement within the peri-implant sulcus. Clinical studies and systematic reviews have associated residual cement with peri-implant inflammation, particularly when restoration margins are positioned submucosally and access for cement removal is limited [5,6,7,8,9,10]. However, cement retention itself is not equivalent to biological exposure to residual cement. The potential effect depends on several factors, including margin position, cement type and amount, abutment geometry, removal technique, and individual susceptibility.

Screw-retained restorations eliminate the risk of residual cement and provide direct retrievability, but they are associated with a different spectrum of prosthetic considerations. Screw-related complications, the position of the access channel, the restorative material used to seal the access opening, and occlusal load distribution may influence their clinical performance. Current systematic reviews and randomized clinical studies generally report comparable survival for cement- and screw-retained restorations, although the types and frequencies of biological and technical complications may differ and radiographic differences are usually small or inconsistent [11,12,13,14,15,16,17].

Interpretation of marginal bone level also requires consideration of contemporary concepts of peri-implant health and disease. Radiographic bone findings alone do not define peri-implant disease and should be interpreted together with clinical inflammatory parameters and changes in supporting bone over time [18,19,20]. This distinction is particularly relevant when very small radiographic differences are detected statistically between prosthetic groups.

The posterior maxilla treated with sinus floor elevation represents a specific clinical setting in which local anatomy, augmentation magnitude, primary stability, loading protocol, and prosthetic planning may jointly influence peri-implant conditions. Long-term studies have demonstrated favorable implant outcomes after maxillary sinus floor elevation, including protocols involving simultaneous implant placement [21,22]. However, the available literature comparing cement- and screw-retained restorations has primarily evaluated broader implant populations and heterogeneous prosthetic settings [2,3,4,11,12,13,14,15,16,17], whereas evidence specifically addressing the relationship between prosthetic retention mode and long-term marginal bone level after maxillary sinus floor elevation remains limited. This represents a clinically relevant knowledge gap because retention-related biological and technical factors are being evaluated in an augmented posterior maxilla, where surgical and prosthetic variables may jointly influence peri-implant bone conditions. The novelty of the present study therefore lies in evaluating the association between definitive prosthetic retention mode and long-term peri-implant marginal bone level specifically within this clinical setting.

Therefore, the primary objective of this study was to evaluate the association between definitive restoration retention mode and peri-implant marginal bone level at a minimum 5-year follow-up after concomitant maxillary sinus floor elevation. Secondary objectives were to examine the association between provisional restoration retention and marginal bone level and to assess whether longitudinal change differed according to definitive retention mode. The null hypothesis was that definitive restoration retention mode would not be associated with peri-implant marginal bone level at the minimum 5-year assessment.

## 2. Materials and Methods

### 2.1. Study Design and Clinical Setting

A retrospective observational cohort study was conducted by analyzing existing clinical and imaging data from the archives of the Implantology Clinic, Faculty of Dental Medicine, Titu Maiorescu University, Bucharest, Romania. Bone-level Dentium SuperLine implants were placed between January 2019 and January 2021 concomitantly with maxillary sinus floor elevation, and the patients underwent annual clinical follow-up. For the present analysis, measurements obtained immediately after implant placement and those corresponding to an available follow-up CBCT examination performed at a minimum of 60 months after placement were extracted retrospectively.

The database was organized at both the patient and implant levels. Radiographic outcomes and prosthetic characteristics were recorded for each implant. Dependence among implants belonging to the same patient was addressed using statistical methods for clustered observations. No a priori sample-size calculation was performed because this retrospective study included all eligible cases available during the predefined study period. The adequacy of the available sample was therefore assessed from the precision of the primary adjusted estimate rather than from a prospective power calculation.

The same clinical cohort was analyzed in a separate study currently under review addressing implant-site preparation techniques. Although the two studies share the underlying clinical and radiographic dataset, the present analysis addresses a distinct research question focused on prosthetic retention mode.

### 2.2. Eligibility Criteria

Eligible participants were adults who received one or more bone-level Dentium SuperLine implants in the posterior maxilla concomitantly with sinus floor elevation and for whom the documentation identified the retention mode of both the provisional and definitive restorations. An initial radiographic assessment and either an available follow-up CBCT examination at a minimum of 60 months after implant placement or documentation of implant loss before this time point were required.

Cases with incomplete clinical or imaging documentation, without a verifiable classification of prosthetic retention, or without either final radiographic follow-up or documentation of implant loss were excluded. Lost implants were not included in the numerical analysis of marginal bone level at the minimum 5-year assessment. Implant losses were retained in the cohort description and analyzed separately as a distinct clinical outcome.

### 2.3. Implant and Prosthetic Protocol

All procedures were performed by the same specialist in oral surgery and implantology. Bone-level Dentium SuperLine implants with diameters of 4.0 or 4.5 mm and lengths of 8, 10, or 12 mm were used. The sinus-elevation approach and implant-site preparation technique were selected according to local anatomy and the need to achieve primary stability. Implant-site preparation was categorized as osteotome, Versah, or Dentium DASK Simple according to the technique recorded for each implant. In the present study, the surgical technique was included only as an adjustment factor.

Provisional poly (methyl methacrylate) restorations were delivered within the first 24–72 h. Immediate restoration without static or dynamic occlusal contacts was used in cases other than All-on-X rehabilitations. Immediate loading with controlled functional contacts was applied exclusively to splinted All-on-X restorations. Definitive restorations were delivered after 6–8 months. The clinical timeline was therefore: implant placement and baseline CBCT at T0, provisional restoration within 24–72 h, definitive restoration at 6–8 months, and final CBCT assessment at ≥60 months after implant placement.

Both provisional and definitive restorations were classified as cement-retained or screw-retained according to the clinical records and prosthetic database. Retention mode was not randomly allocated but resulted from the clinical and prosthetic decision made for each case. The database did not contain uniform information on cement type, definitive restorative material, abutment material, cementation-margin position, emergence profile, or the protocol used to remove residual cement.

### 2.4. Radiographic Assessment

Peri-implant marginal bone level was measured on CBCT examinations obtained immediately after implant placement and on an available follow-up CBCT examination performed at a minimum of 60 months after placement. For brevity, this assessment is referred to as the 5-year follow-up throughout the manuscript. The examinations used in the present analysis were obtained from the patients’ existing imaging records and were not acquired additionally for the purposes of this retrospective study. The immediate post-implantation CBCT examination was considered the radiographic baseline for the longitudinal assessment. Because definitive restorations were delivered after 6–8 months, the interval from baseline to the final examination encompassed both the provisional and definitive prosthetic phases. Both assessments were performed in the same clinic using the same Veraview X800 CBCT unit (J. MORITA Corp., Kyoto, Japan). The acquisition protocol used a field of view of 80 × 80 mm, a voxel size of 0.125 mm, 80 kV, 6 mA, an exposure time of 9.4 s, and a 180° rotation. DICOM volumes were analyzed using Romexis software 7 (Version 6.5.3). Because the present study was based on retrospectively available clinical imaging, the exact reconstruction, windowing, and artifact-reduction settings were not systematically recorded, and the use of a dedicated metal artifact reduction algorithm could not be verified.

The volume was reoriented according to the longitudinal axis of each implant before measurement. On the mesial and distal aspects, the linear distance from the implant shoulder to the most coronal radiographically visible bone-to-implant contact was measured. A value of 0 mm was assigned when the most coronal bone-to-implant contact coincided with the implant shoulder. The value used for statistical analysis was the arithmetic mean of the mesial and distal measurements, with higher values indicating a more apical position of the bone-to-implant contact.

Three clinicians experienced in implantology and CBCT interpretation evaluated the examinations. No formal examiner calibration exercise was documented before the retrospective assessment, and blinding to prosthetic retention mode was not formally documented. When disagreements occurred regarding landmark identification or measurement, the images were jointly reassessed and the final value was established by consensus. No formal intra- or inter-examiner repeatability analysis was performed; therefore, the measurement uncertainty associated with observer-dependent landmark identification could not be quantified.

### 2.5. Exposures, Outcomes, and Covariates

The primary exposure was the retention mode of the definitive restoration: cement-retained or screw-retained. The retention mode of the provisional restoration was considered the secondary exposure.

The primary outcome was marginal bone level at the minimum 5-year assessment, expressed in millimeters. The secondary outcome was the change in marginal bone level from baseline. Longitudinal change was calculated as the value obtained at the minimum 5-year assessment minus the baseline value; a positive value indicated apical displacement of the bone-to-implant contact. This longitudinal outcome represented the overall marginal bone change from implant placement to final follow-up and was not interpreted as bone change occurring exclusively during exposure to the definitive restoration.

Available covariates were age, female/male category, number of implants per patient, smoking, presence of periodontitis, implant-site preparation technique, sinus elevation height, and the immediate restoration or immediate loading protocol. Sinus elevation height was defined as the vertical distance between the original maxillary sinus floor and the most apical point of the elevated sinus floor, measured along the implant axis on the immediate postoperative CBCT examination. Although the clinical protocol documented the diameter and length ranges used in the cohort, implant-specific diameter and length values were not available as analyzable variables in the statistical dataset. Exact implant position/tooth site, definitive prosthetic restoration type, and single-versus-splinted configuration were likewise unavailable as analyzable implant-level variables and therefore could not be included in the between-group comparisons or adjusted models.

Smoking status and the presence of periodontitis were obtained from the clinical documentation available at the time of implant treatment and were analyzed as binary variables according to the patient records. The database did not allow differentiation between current and former smokers or retrospective reclassification of periodontitis according to a staging and grading system.

### 2.6. Statistical Analysis

Analyses were performed using IBM SPSS Statistics for Windows, version 32.0. Quantitative variables were described using the mean, standard deviation, median, interquartile range, and minimum-maximum range, whereas categorical variables were summarized as absolute frequencies and percentages.

The unit of analysis was specified according to the type of analysis. Overall patient characteristics reported for the cohort were summarized using one observation per patient. Comparisons according to definitive retention mode were performed at the implant level because retention mode and radiographic outcomes were defined for individual implants. Accordingly, patient-level covariates included in implant-level descriptive comparisons were repeated across implants belonging to the same patient. In adjusted implant-level analyses, within-patient dependence was accounted for by clustering on patients. Implant loss was likewise analyzed at the implant level.

Comparability between implants with definitive cement-retained and screw-retained restorations was assessed descriptively using the standardized mean difference (SMD), reported as an absolute value. Values < 0.10 were considered negligible imbalances, values between 0.10 and 0.20 small imbalances, and values > 0.20 relevant imbalances. The relationship between provisional and definitive restoration retention modes was assessed using the chi-square test and Fisher’s exact test, and the magnitude of association was quantified using Phi/Cramer’s V. Potential collinearity between the two prosthetic variables was evaluated using the variance inflation factor (VIF) and tolerance.

The distribution of marginal bone level at the minimum 5-year assessment was positively skewed and included a large proportion of 0 mm values. As a complementary analysis of the outcome distribution, the proportions of implants with values of 0 mm and >0 mm were compared between definitive retention modes using the chi-square test and Fisher’s exact test, and the magnitude of association was expressed using the Phi coefficient. Unadjusted comparisons between the two retention modes primarily used the Mann–Whitney U test. Welch’s *t*-test was additionally reported to estimate the unadjusted difference between means. Effect size was expressed using the rank-biserial correlation coefficient and Hedges’ g. Unadjusted comparisons were considered exploratory and were interpreted alongside the primary adjusted GEE model, which accounted for dependence among implants belonging to the same patient.

The primary adjusted analysis used generalized estimating equations (GEE) [23], with a Gaussian distribution, identity link, exchangeable working correlation structure, and robust standard errors. The implant was the analytical unit, with the patient used as the clustering unit to account for multiple implants contributed by the same patient. The dependent variable was peri-implant marginal bone level at the minimum 5-year assessment, and the main predictor was definitive screw-retained versus cement-retained restoration. The model was adjusted for baseline marginal bone level, age, female/male category, smoking, periodontitis, number of implants per patient, implant-site preparation technique, sinus elevation height, and the immediate loading versus immediate restoration protocol.

Because provisional and definitive retention modes were highly correlated, provisional retention was not included in the primary adjusted model. As a sensitivity analysis, the primary model was repeated with provisional retention included. An additional sensitivity analysis was restricted to implants that maintained the same retention strategy from the provisional to the definitive restoration.

To address the marked concentration of observations at 0 mm and the right-skewed distribution of positive marginal bone-level values, an additional two-part sensitivity approach was evaluated [24]. The first component specified a binary logistic GEE to model the probability of a marginal bone level > 0 mm versus 0 mm. The second component was restricted to implants with values > 0 mm and used a Gamma GEE with a log link to model the magnitude of positive marginal bone-level values. Both components were specified with the implant as the analytical unit, the patient as the clustering unit, an exchangeable working correlation structure, robust standard errors, and the same covariates as the primary GEE model.

Adjusted marginal means for the two definitive retention modes were derived from the model using predictive standardization.

Overall change in marginal bone level from the immediate post-implantation baseline to final follow-up was evaluated using a linear mixed model that included the two radiographic assessment time points. The model included assessment time, definitive retention mode, and the time × definitive retention interaction, together with the same clinical, surgical, and prosthetic covariates. Random components were included for the patient and for implants nested within patients, and estimation was performed by maximum likelihood. The time × definitive retention interaction was the main parameter used to assess a potential difference between the longitudinal trajectories of the two groups.

Sensitivity of the primary result to the estimation method was evaluated using multivariable linear regression with robust standard errors clustered by patient, using the same dependent variable, main predictor, and covariates as the GEE model. Statistical significance was set at *p* < 0.05, and interpretation integrated confidence intervals, effect size, and the clinical magnitude of the differences. Reporting followed the STROBE principles [25].

There were no missing values for the covariates included in the primary models. The radiographic analyses included 522 of the 528 implants, from 247 of the 248 patients. The six implants lost before the final assessment could not contribute to the radiographic outcome and were excluded from these analyses; the complete-case approach resulted in no additional exclusions. Implant losses were analyzed separately, descriptively and using Fisher’s exact test, given the small number of events. No imputation of the 5-year marginal bone level was performed for lost implants because a valid final radiographic measurement was not available after implant loss.

### 2.7. Ethical Considerations

The study was conducted in accordance with the Declaration of Helsinki and was approved by the Ethics Committee of Titu Maiorescu University, Bucharest, through Decision No. 3 of 20 January 2017. Informed consent was obtained in accordance with institutional requirements. This approval covered the clinical protocol and patient monitoring from which the data analyzed retrospectively in the present study were derived.

## 3. Results

### 3.1. Cohort Characteristics

The cohort included 248 patients and 528 bone-level Dentium SuperLine implants. The retrospective database contained the eligible cohort used for analysis; a separate screening log allowing quantification and classification of cases excluded before cohort assembly was not available. At the patient level, the mean age was 42.00 ± 10.66 years; 156 patients (62.9%) were in the female category, 77 (31.0%) were smokers, and 47 (19.0%) had periodontitis. Definitive cement-retained restorations were used for 162 implants from 70 patients, whereas definitive screw-retained restorations were used for 366 implants from 178 patients. Of the 528 implants, 6 (1.1%) were lost before the final assessment and therefore could not contribute a valid final marginal bone-level measurement. The remaining 522 implants were radiographically evaluable and belonged to 247 of the original 248 patients. The reduction in the number of patients reflects the absence of an evaluable final radiographic outcome for one patient after implant loss. Three of the 162 implants with definitive cement-retained restorations were lost (1.9%), compared with 3 of the 366 implants with definitive screw-retained restorations (0.8%). The difference was not statistically significant according to Fisher’s exact test (*p* = 0.377). No additional final CBCT examinations were missing; thus, the six implants without final radiographic measurements were exactly the six implants lost before the final assessment.

In the implant-level descriptive analysis, the two groups had similar values for most demographic and clinical characteristics. Baseline marginal bone level was slightly higher in the cement-retained group, with a small imbalance according to the SMD. The most evident difference between groups was the retention mode of the provisional restoration. A smaller but relevant imbalance was also observed for the number of implants per patient. The remaining covariates showed small or negligible differences. Comparative characteristics of the two groups and SMD values are presented in Table 1.

### 3.2. Definitive Restoration Retention

The radiographic analysis included 159 implants with definitive cement-retained restorations and 363 with definitive screw-retained restorations. The mean was 0.133 ± 0.184 mm in the cement-retained group and 0.105 ± 0.172 mm in the screw-retained group. The median was 0 mm in both groups. A value of 0 mm was observed in 59.1% of implants with cement-retained restorations and 67.5% of those with screw-retained restorations. Correspondingly, values > 0 mm were observed in 40.9% of implants with cement-retained restorations and 32.5% of those with screw-retained restorations. The difference in the proportions of 0 mm and >0 mm values was not statistically significant (chi-square = 3.41; *p* = 0.065; Fisher’s exact test *p* = 0.073), and the magnitude of association was very small (Phi = 0.08).

The Mann–Whitney test did not identify a statistically significant difference (U = 31,366; *p* = 0.064). The effect size was small (rank-biserial r = 0.09). The complementary parametric analysis indicated an unadjusted mean difference of approximately 0.03 mm between cement-retained and screw-retained restorations (95% CI: 0.00–0.06 mm; *p* = 0.102), with Hedges’ g = 0.16.

### 3.3. Association Between Provisional and Definitive Restoration Retention

The retention mode of the provisional restoration was very strongly associated with definitive restoration retention. Of the 175 implants with a cement-retained provisional restoration, 160 (91.4%) subsequently received a definitive cement-retained restoration and 15 (8.6%) a screw-retained restoration. Of the 353 implants with a screw-retained provisional restoration, 351 (99.4%) subsequently received a definitive screw-retained restoration and only two (0.6%) a cement-retained restoration. The distribution of definitive retention mode according to provisional restoration retention is presented in Table 2.

The association was statistically significant and very strong (chi-square(1) = 454.17; *p* < 0.001; Phi/Cramer’s V = 0.927). Collinearity assessment yielded VIF = 7.15 and tolerance = 0.140. The strong association between provisional and definitive retention reflected substantial continuity of the prosthetic strategy. Accordingly, coefficients obtained when both variables were included in the adjusted model were interpreted conditionally, and no independent biological effect was attributed to provisional retention.

### 3.4. Provisional Restoration Retention

Among the evaluable implants, 172 had cement-retained provisional restorations and 350 had screw-retained provisional restorations. Mean marginal bone level was 0.13 ± 0.18 mm and 0.11 ± 0.17 mm, respectively. The difference was not statistically significant according to the Mann–Whitney test (U = 32,165; *p* = 0.135) and had a small effect size. The proportion of 0 mm values was 60.5% in the cement-retained group and 67.1% in the screw-retained group, with no significant difference according to Fisher’s exact test (*p* = 0.144). Unadjusted comparative results for definitive and provisional retention are summarized in Table 3.

### 3.5. Adjusted Analysis of Marginal Bone Level at the Minimum 5-Year Follow-Up

The primary GEE model included 522 implants from 247 patients. After adjustment for baseline marginal bone level and the available demographic, clinical, surgical, and prosthetic covariates, definitive screw-retained restoration was not significantly associated with marginal bone level at the minimum 5-year follow-up (B = −0.0028 mm; robust SE = 0.0070; 95% CI: −0.0165–0.0108; *p* = 0.684). The absolute adjusted between-group difference was therefore approximately 0.003 mm.

The model used a Gaussian distribution with an identity link, exchangeable working correlation structure, and robust standard errors. The exchangeable working correlation parameter was 0.120. QIC and corrected QIC values were 534.32 and 534.93, respectively. Coefficients from the primary GEE model are presented in Table 4.

Apart from baseline marginal bone level, the number of implants per patient and the immediate loading protocol were the only covariates significantly associated with the outcome in the primary GEE model. Each additional implant per patient was associated with an estimated 0.0099 mm increase in marginal bone level at follow-up (95% CI: 0.0032–0.0167; *p* = 0.004), whereas immediate loading was associated with an estimated value 0.0185 mm lower than immediate restoration (95% CI: −0.0346–−0.0023; *p* = 0.025). These secondary associations should be interpreted cautiously because they were not the primary focus of the study.

Adjusted marginal means derived from the GEE model are presented in Table 5.

Adjusted marginal means were 0.115 mm for cement-retained restorations and 0.112 mm for screw-retained restorations. The adjusted difference was −0.0028 mm (95% CI: −0.0165–0.0108; *p* = 0.684), indicating no statistically significant association between definitive retention mode and marginal bone level in the primary adjusted model. Baseline marginal bone level remained the strongest predictor of the final value (B = 1.0585; *p* < 0.001).

### 3.6. Longitudinal Analysis According to Definitive Retention

The longitudinal model included 522 implants from 247 patients, with each implant contributing two measurements, at baseline and at 5 years. The data were organized in longitudinal format, yielding 1044 observations, two for each implant. The model was estimated by maximum likelihood and included a random intercept at the patient level and a random component for implants nested within patients. The model converged, with AIC = −1843.552, BIC = −1764.339, and log-likelihood = 937.776. The estimated variance of the patient-level intercept was 0.0078, the component corresponding to implants nested within patients was 0.0164, and the residual variance was 0.0020. In the definitive cement-retained group, the adjusted increase in marginal bone level was 0.030 mm (95% CI: 0.020–0.040; *p* < 0.001), whereas in the screw-retained group it was 0.023 mm (95% CI: 0.016–0.030; *p* < 0.001).

Adjusted longitudinal contrasts for the two retention modes are presented in Table 6.

Estimated adjusted marginal values were 0.1002 mm at baseline and 0.1302 mm at follow-up for cement-retained restorations, compared with 0.0832 mm and 0.1062 mm, respectively, for screw-retained restorations. The adjusted change was 0.030 mm in the cement-retained group and 0.023 mm in the screw-retained group. The time × definitive retention interaction, which quantifies the difference between these changes, was not statistically significant (B = −0.007 mm; 95% CI: −0.019–0.005; *p* = 0.249). Thus, although both groups showed a small adjusted apical displacement of the bone-to-implant contact during follow-up, the longitudinal trajectories did not differ significantly between the two retention modes.

These trajectories describe overall marginal bone change from implant placement to final follow-up within groups defined by definitive retention mode; they should not be interpreted as changes occurring exclusively after delivery of the definitive restoration.

### 3.7. Sensitivity Analyses

Several sensitivity analyses were performed to evaluate the robustness of the primary finding and the consequences of the strong association between provisional and definitive retention modes.

When provisional retention was added to the primary GEE model, the coefficient for definitive screw-retained versus cement-retained restoration changed from −0.0028 mm (95% CI: −0.0165–0.0108; *p* = 0.684) to −0.0194 mm (95% CI: −0.0385–−0.0004; *p* = 0.045). In the same model, provisional screw retention had a coefficient of 0.0177 mm (95% CI: −0.0003–0.0356; *p* = 0.054). Given the very strong association between provisional and definitive retention modes (Phi/Cramer’s V = 0.927; VIF = 7.15; tolerance = 0.140), the marked change in the definitive-retention coefficient indicates instability when both highly collinear prosthetic variables are entered simultaneously.

A further GEE analysis restricted to the 505 implants from 240 patients who maintained the same retention strategy from the provisional to the definitive restoration yielded a coefficient of −0.0021 mm for screw-retained versus cement-retained restoration (robust SE = 0.0072; 95% CI: −0.0162–0.0119; *p* = 0.766). Seventeen implants changed retention strategy: 15 from cement-retained provisional to screw-retained definitive restoration and two from screw-retained provisional to cement-retained definitive restoration. Because of the small and markedly imbalanced number of changes, no separate multivariable model was fitted to this subgroup.

The cluster-robust multivariable linear regression using the same covariates as the primary GEE model also showed no significant association between definitive retention mode and final marginal bone level (B = −0.0027 mm; robust SE = 0.0072; 95% CI: −0.0169–0.0115; *p* = 0.707).

For the two-part sensitivity approach, the binary logistic GEE component could not be validly estimated because baseline marginal bone level produced complete or quasi-complete separation: all 147 implants with a baseline marginal bone level >0 mm also had a final value >0 mm. The Gamma GEE component was estimable in 183 implants with positive final marginal bone-level values from 119 patients. Among these implants, definitive retention mode was not associated with the magnitude of positive marginal bone level (B = 0.0073; Exp(B) = 1.007; 95% CI for Exp(B): 0.915–1.109; *p* = 0.881).

The estimates for definitive retention obtained across the primary and sensitivity analyses are summarized in Table 7.

The primary GEE, the concordant-strategy analysis, and the cluster-robust regression produced very similar estimates, all close to zero and statistically nonsignificant. In contrast, simultaneous inclusion of provisional and definitive retention produced a larger coefficient for definitive retention. This discrepancy indicates sensitivity to model specification in the presence of the very strong collinearity between the two retention variables.

## 4. Discussion

The present study did not identify a significant adjusted association between definitive prosthetic retention mode and marginal bone level at the minimum 5-year follow-up when the primary model was specified without provisional retention. Although the unadjusted mean marginal bone level was numerically higher for cement-retained than for screw-retained restorations, the unadjusted comparison was not statistically significant. In the primary patient-clustered GEE model, the adjusted difference between screw-retained and cement-retained restorations was only −0.0028 mm (95% CI: −0.0165–0.0108; *p* = 0.684). The adjusted marginal means were correspondingly similar, at 0.115 mm and 0.112 mm, respectively.

The sensitivity analyses are important for evaluating the influence of model specification on the estimated association between retention modes. When provisional and definitive retention were entered simultaneously, the coefficient for definitive retention increased to −0.0194 mm and reached the conventional threshold for statistical significance. However, provisional and definitive retention were extremely strongly associated (Phi/Cramer’s V = 0.927; VIF = 7.15), and the substantial change in the definitive-retention coefficient indicates instability related to simultaneous modeling of these highly collinear exposures. In contrast, the analysis restricted to implants that maintained the same retention strategy yielded an adjusted difference of only −0.0021 mm (*p* = 0.766), and cluster-robust regression produced a nearly identical estimate of −0.0027 mm (*p* = 0.707). Taken together, these analyses do not support a robust association between definitive retention mode and final marginal bone level.

The longitudinal analysis reached the same clinical conclusion. Marginal bone level increased slightly over time in both groups, but the time × definitive retention interaction was not statistically significant (B = −0.0069 mm; 95% CI: −0.0187–0.0049; *p* = 0.249). Thus, the study did not detect a difference in longitudinal marginal bone change between the two retention strategies. Because definitive restorations were delivered 6–8 months after implant placement, the longitudinal interval encompassed both the provisional and definitive prosthetic phases and cannot be attributed exclusively to definitive retention mode.

The primary adjusted analysis did not reject the null hypothesis of no association between definitive retention mode and marginal bone level at follow-up. However, the absence of a statistically significant association should not be interpreted as evidence of equivalence or non-inferiority between retention strategies. The study was not designed for either purpose, and no equivalence or non-inferiority margin was prespecified. Interpretation should therefore place greater emphasis on the magnitude of the estimated association and consistency across analyses than on a binary classification of the result as “significant” or “non-significant”.

Because no clinically meaningful between-group difference was prespecified, the study cannot be claimed to have been prospectively powered for a specific clinically meaningful between-group difference. The 95% confidence interval around the primary adjusted estimate had a width of approximately 0.027 mm, corresponding to an approximate precision of ±0.014 mm.

Previous literature comparing cement-retained and screw-retained restorations is not uniform. Several prospective and randomized studies have reported small or no differences in peri-implant bone outcomes between the two retention strategies [12,13,14,15,26,27], whereas systematic reviews and clinical studies have identified differences in biological complication profiles and have continued to recognize residual cement as a relevant local risk factor around cement-retained restorations [5,6,7,8,9,10,16,17,28,29,30,31]. These apparently divergent findings should be interpreted in the context of substantial heterogeneity in restoration type, prosthetic design, implant position, cementation procedures, and follow-up duration [2,3,4]. Accordingly, the findings of the present study should be viewed within this heterogeneous evidence base rather than as confirmation of uniformly similar biological behavior between the two retention strategies. A more recent cohort study extended the comparison to angulated screw-retained versus cement-retained crowns at 5 years, illustrating that retention mode continues to be evaluated in relation to the prosthetic context and implant position rather than as an isolated biological determinant [28].

A relevant methodological feature of the present study is the difference between the crude and adjusted comparisons. Baseline marginal bone level was slightly higher in the cement-retained group and was the strongest predictor of the 5-year value. Its coefficient, close to one, indicates strong continuity between baseline and final radiographic status. Including baseline in the model therefore changes the estimated relationship between retention and the final value. This finding supports the use of a model adjusted for the baseline value and shows why a simple comparison of 5-year means cannot be the sole basis for interpreting the difference between the two groups. Retention mode was also not randomly allocated, so statistical adjustment cannot fully eliminate confounding by indication. Prosthetic factors such as implant position and angulation, abutment configuration, restoration design, and screw-access channel position may contribute both to the choice of retention mode and to local peri-implant conditions. Because these characteristics were not uniformly available in the database, residual confounding cannot be excluded. Propensity-score methods would likewise balance only measured covariates and could not account for these undocumented determinants of retention selection.

The very strong association between provisional and definitive restoration retention is another important feature of the cohort. Nearly all implants maintained the same retention strategy from the provisional stage to the definitive restoration, and a Phi/Cramer’s V of 0.927, VIF of 7.15, and low tolerance confirm substantial overlap between the two variables. From a clinical perspective, this continuity is plausible because the implant axis, prosthetic space, and restoration strategy established during the initial phase tend to influence the definitive restoration as well.

The strong association between provisional and definitive retention therefore largely reflected continuity of the prosthetic treatment strategy rather than two unrelated exposures. This substantial collinearity limits the ability to estimate separate associations for the provisional and definitive prosthetic phases and may affect the stability of their individual coefficients.

Given the very strong correlation between provisional and definitive retention, provisional retention was excluded from the primary adjusted model and evaluated in sensitivity analysis.

Residual cement is a recognized local factor around cement-retained restorations, although cement retention itself is not equivalent to peri-implant disease [5,6,7,8,9,10,29,30,31]. Because cement type, amount, margin position, and the actual presence of residual cement were not recorded, the observed radiographic difference cannot be attributed to this mechanism. Because these cement-related variables were not evaluated, the present findings neither confirm nor refute the biological risks associated with residual cement reported in previous studies. Abutment geometry, cementation technique, and other prosthetic characteristics may also influence residual cement or peri-implant conditions [32,33,34,35,36,37,38,39], but these variables were not uniformly available in the present database.

From this perspective, the present study should be interpreted primarily as an observational comparison of two prosthetic strategies used in clinical practice rather than as evidence attributable specifically to cementation or screw retention. This distinction is also supported by prospective studies showing that bone changes after prosthesis delivery may be influenced by characteristics of the implant-abutment-restoration complex and local conditions, even in the absence of a major difference in survival [40].

The longitudinal analysis is particularly important for the clinical interpretation of these findings. Adjusted marginal bone-level change was 0.030 mm in the cement-retained group and 0.023 mm in the screw-retained group, with a between-group difference of only 0.007 mm that was not statistically significant (*p* = 0.249). Thus, the study did not detect a statistically significant difference in longitudinal marginal bone change between the two retention strategies; this finding should not be interpreted as evidence of equivalence. No equivalence or non-inferiority margin was prespecified.

The sensitivity analyses clarify the influence of model specification on the estimated association. The cluster-robust regression and the GEE analysis restricted to concordant retention strategies produced estimates that were very close to the primary GEE estimate and were statistically nonsignificant. In contrast, simultaneous inclusion of provisional and definitive retention produced a larger coefficient that reached the conventional threshold for statistical significance. Given the very strong collinearity between these two retention variables, this discrepancy should be interpreted as model-specification instability rather than as evidence of a clinically relevant association.

The magnitude of the primary adjusted difference should also be considered in relation to the radiographic method. The absolute primary adjusted difference was approximately 0.003 mm, substantially smaller than the CBCT voxel size of 0.125 mm. Even the larger −0.0194 mm estimate obtained only when provisional and definitive retention were entered simultaneously remained well below the nominal voxel dimension. Intra- and inter-examiner reproducibility was not quantified; therefore, differences of this magnitude cannot be interpreted confidently as anatomical differences at the individual-implant level. Experimental literature on CBCT around implants shows that peri-implant bone measurements may be influenced by bone thickness, defect size, field of view, and artifacts generated by implant material [41,42,43]. Gurjar et al. showed that implants and implant-supported prostheses can generate artifacts that affect the accuracy of CBCT assessment in peri-implant regions [41]. da Fonte et al. found that the number of zirconia implants and reconstruction thickness did not significantly influence the detection of 3 mm buccal peri-implant bone defects, although diagnostic performance was numerically lower when two implants were present [42]. Almohandes et al. compared radiographic measurements with reference assessments under experimental conditions [43]. In this context, differences of only a few thousandths to hundredths of a millimeter should not be presented as definite anatomical changes at the level of an individual implant, irrespective of whether a particular model reaches a conventional significance threshold.

The context of sinus floor elevation is relevant because prosthetic outcomes were evaluated in a selected population of implants placed in the augmented posterior maxilla. Clinical studies with 5-year or longer follow-up have documented the possibility of stable outcomes after sinus floor elevation and implant placement, including protocols with simultaneous placement in selected cases [21,22]. However, these findings do not allow bone behavior to be attributed to prosthetic retention mode. In the present GEE model, sinus elevation height was not independently associated with marginal bone level at 5 years after inclusion of the baseline value, supporting its role primarily as an adjustment factor in the prosthetic question examined here.

The importance of the baseline bone value extends beyond its role as a simple statistical covariate. In a prospective cohort with 10-year follow-up, early peri-implant bone changes were associated with subsequent bone stability and the risk of developing peri-implantitis [44]. These data support interpretation of baseline bone level and early remodeling as relevant elements in long-term outcome assessment and justify inclusion of the baseline value in models comparing prosthetic strategies. In our study, the strong association between baseline bone level and the 5-year value indicates that a substantial proportion of the final variation was already present before the prosthetic follow-up period analyzed. Baseline marginal bone level represented the initial radiographic distance between the implant shoulder and the marginal bone-to-implant contact and was therefore included in the adjusted model. However, this measurement is not equivalent to a dedicated assessment of the vertical position of the implant shoulder relative to the alveolar crest, and crestal versus subcrestal insertion depth was not available as a separate variable. Other implant-abutment variables, including repeated abutment manipulation, platform configuration, abutment height, implant depth, and emergence angle, have also been evaluated in relation to marginal bone remodeling [45,46,47,48,49,50,51,52,53], but these factors were not uniformly recorded in the present cohort.

Among the other covariates, the number of implants per patient and the immediate loading protocol showed significant associations in the GEE model. These findings should be considered secondary and exploratory. The number of implants per patient may reflect rehabilitation complexity and shared patient-level characteristics not fully captured by the available covariates. In addition, immediate loading in this cohort was used in the context of splinted All-on-X rehabilitations, whereas immediate restoration represented a different clinical protocol. The negative coefficient associated with immediate loading should therefore not be interpreted as evidence that immediate loading protects marginal bone. The two protocols were not randomly assigned and represent different therapeutic contexts.

Strengths of the study include the number of implants analyzed, a minimum follow-up of 5 years, availability of baseline and final measurements, use of the same implant system and CBCT unit, and modeling of dependence among implants belonging to the same patient. The analytical sample remained constant across the primary models, and the absence of additional missing covariate values limits the risk that differences between estimates were driven by changes in the analyzed population. The use of unadjusted comparisons, an adjusted GEE model, a longitudinal model, and several complementary sensitivity analyses allowed the main research question to be examined from different statistical perspectives.

The main limitations are the retrospective, single-center design and the absence of random allocation of retention mode. Retention mode was selected clinically, and the factors determining this choice were not uniformly documented. Therefore, the multivariable adjustment can reduce confounding from the measured covariates but cannot eliminate confounding by indication or residual confounding.

The marginal-bone analyses were also restricted to implants retained until the final radiographic assessment. Although only six implants were lost and loss rates did not differ significantly between retention groups, exclusion of these implants may introduce a small degree of survivorship bias if implants that failed would otherwise have shown less favorable marginal bone outcomes. Because the retrospective database did not include a screening log for cases excluded before assembly of the analytical cohort, the number and retention-mode distribution of such exclusions could not be reconstructed. Consequently, selection or attrition bias related to pre-inclusion exclusions cannot be quantified.

The very high collinearity between provisional and definitive retention limits the ability to estimate separate associations for the two prosthetic phases. Uniform information was not available on definitive restorative material, abutment type and height, cementation-margin position, cement type and amount, emergence profile and angle, screw-access channel position, occlusal scheme, or maintenance interventions. In addition, implant-specific diameter and length values, exact implant position/tooth site, definitive prosthetic restoration type, and single-versus-splinted configuration were not available as analyzable implant-level variables in the statistical dataset and therefore could not be compared between retention groups. These unavailable implant-abutment and prosthetic characteristics represent potential sources of residual confounding. Clinical peri-implant measurements such as bleeding on probing and probing depth were also not included, and intra- and inter-examiner reproducibility of radiographic measurements was not quantified. Smoking and periodontitis were available only as recorded in the clinical documentation, without uniform information on intensity of tobacco exposure or periodontitis severity and classification. The absolute primary adjusted between-group difference was approximately 0.003 mm, substantially smaller than the nominal CBCT voxel dimension of 0.125 mm. Even the larger approximately 0.019 mm estimate obtained in the sensitivity model including both highly collinear retention variables remained well below the voxel dimension. Accordingly, differences of this magnitude cannot be confidently distinguished from measurement uncertainty at the individual-implant level. The timing of the final radiographic assessment was not uniformly fixed at exactly 60 months but corresponded to an available CBCT examination after this threshold had been reached, which may introduce some heterogeneity in the actual follow-up duration.

From a clinical perspective, the findings do not justify a universal rule favoring either retention mode. Screw retention provides retrievability and avoids the problem of residual cement, whereas cement retention may remain useful when implant position and axis, restoration design, or occlusal requirements limit favorable screw access [1,2]. In the analyzed cohort, the primary adjusted analysis did not identify a significant association between definitive retention mode and marginal bone level, and longitudinal marginal bone change did not differ significantly between the two strategies. Retention mode should therefore be integrated into a broader prosthetic decision that considers implant position, access for maintenance, cement control, transmucosal profile, restoration design, and patient-specific clinical factors.

Furthermore, because the cohort comprised a single implant system placed exclusively in the posterior maxilla in conjunction with sinus floor elevation, the findings should not be generalized directly to other implant systems, mandibular sites, or non-augmented implant treatment.

## 5. Conclusions

In this retrospective cohort of implants placed concomitantly with maxillary sinus floor elevation, definitive prosthetic retention mode was not significantly associated with marginal bone level at the minimum 5-year follow-up in the primary adjusted analysis. The absolute adjusted difference between screw-retained and cement-retained restorations was approximately 0.003 mm. Longitudinal marginal bone change also did not differ significantly between the two retention modes.

Sensitivity analyses showed that the estimated association was influenced by the strong collinearity between provisional and definitive retention, whereas analyses excluding this simultaneous adjustment, including the concordant-strategy analysis and cluster-robust regression, consistently showed negligible differences. Given the observational design, residual confounding, and radiographic measurement limitations, the findings should not be interpreted as evidence of causal effects, clinical superiority, or equivalence of either retention strategy. Retention mode should therefore be selected according to the broader prosthetic and clinical context.

## Figures and Tables

**Table 1:** Implant-level cohort characteristics according to definitive restoration retention mode.

Variable	Cement-Retained Restoration (*n* = 162; 70 Patients)	Screw-Retained Restoration (*n* = 366; 178 Patients)	Absolute SMD
Baseline marginal bone level, mm, mean ± SD; median (IQR)	0.10 ± 0.17; 0.00 (0.00–0.21)	0.08 ± 0.15; 0.00 (0.00–0.16)	0.138
Age, years, mean ± SD; median (IQR)	44.01 ± 9.65; 46.00 (38.00–50.75)	44.23 ± 10.36; 45.00 (37.00–52.00)	0.022
Total number of implants per patient, mean ± SD; median (IQR)	2.78 ± 0.98; 3.00 (2.00–4.00)	2.53 ± 1.02; 2.00 (2.00–3.00)	0.246
Sinus elevation height, mm	3.02 ± 1.18	3.04 ± 1.29	0.014
Cement-retained provisional restoration	160 (98.8%)	15 (4.1%)	2.011
Screw-retained provisional restoration	2 (1.2%)	351 (95.9%)	
Female category	97 (59.9%)	237 (64.8%)	0.101
Male category	65 (40.1%)	129 (35.2%)	
Smoker	59 (36.4%)	122 (33.3%)	0.065
Periodontitis	30 (18.5%)	83 (22.7%)	0.101
Osteotome	62 (38.3%)	142 (38.8%)	0.119
Versah	47 (29.0%)	124 (33.9%)	
Dentium DASK Simple	53 (32.7%)	100 (27.3%)	
Immediate restoration	139 (85.8%)	311 (85.0%)	0.023
Immediate loading	23 (14.2%)	55 (15.0%)	

Note: SMD, standardized mean difference. The table is based on implant-level analysis. For variables defined at the patient level, namely age, female/male category, smoking, periodontitis, and total number of implants per patient, the same value was assigned to all implants belonging to that patient. Therefore, the descriptive statistics and corresponding SMD values for these variables are weighted at the implant level and do not represent patient-level descriptive estimates. Radiographic values were calculated for implants with available measurements.

**Table 2:** Association between provisional and definitive restoration retention modes.

Provisional Restoration	Definitive Cement-Retained	Definitive Screw-Retained	Total
Cement-retained	160 (91.4%)	15 (8.6%)	175
Screw-retained	2 (0.6%)	351 (99.4%)	353
Total	162	366	528

**Table 3:** Peri-implant marginal bone level at 5 years according to retention mode.

Exposure	n	Mean ± SD, mm	Median [IQR], mm	0 mm Value	*p*
Definitive cement-retained	159	0.133 ± 0.184	0.000 [0.000–0.300]	94 (59.1%)	0.064
Definitive screw-retained	363	0.105 ± 0.172	0.000 [0.000–0.210]	245 (67.5%)	
Provisional cement-retained	172	0.130 ± 0.180	0.000 [0.000–0.300]	104 (60.5%)	0.135
Provisional screw-retained	350	0.110 ± 0.170	0.000 [0.000–0.210]	235 (67.1%)	

*p*-values correspond to the Mann–Whitney U test comparing marginal bone level between the two categories of each exposure. IQR, interquartile range.

**Table 4:** Adjusted GEE model for peri-implant marginal bone level at 5 years.

Predictor	B, mm	Robust SE	95% CI	*p*
Definitive screw-retained vs. cement-retained	−0.0028	0.0070	−0.0165–0.0108	0.684
Baseline marginal bone level, mm	1.0585	0.0181	1.0231–1.0940	<0.001
Age, years	−0.0002	0.0003	−0.0008–0.0003	0.362
Male vs. female category	0.0094	0.0059	−0.0021–0.0209	0.108
Smoker vs. non-smoker	−0.0118	0.0061	−0.0237–0.0002	0.053
Periodontitis: present vs. absent	0.0012	0.0084	−0.0153–0.0177	0.887
Number of implants per patient	0.0099	0.0034	0.0032–0.0167	0.004
Versah vs. osteotome	0.0097	0.0061	−0.0023–0.0218	0.114
DASK Simple vs. osteotome	0.0075	0.0074	−0.0069–0.0219	0.308
Sinus elevation height, per mm	0.0006	0.0024	−0.0041–0.0054	0.795
Immediate loading vs. immediate restoration	−0.0185	0.0083	−0.0346–−0.0023	0.025

Note: Gaussian GEE model with identity link, exchangeable working correlation structure, robust standard errors, and patient as the clustering unit. The reference category for definitive retention was the cement-retained restoration.

**Table 5:** Adjusted marginal means of marginal bone level at 5 years according to definitive restoration retention.

Definitive Restoration	Adjusted Mean, mm	SE	95% CI
Cement-retained	0.1153	0.0059	0.1037–0.1268
Screw-retained	0.1124	0.0034	0.1057–0.1192
Difference, screw-retained vs. cement-retained	−0.0028	0.0070	−0.0165–0.0108

**Table 6:** Adjusted longitudinal change in marginal bone level according to definitive retention.

Contrast	B, mm	SE	95% CI	*p*
Change, cement-retained restoration	0.0299	0.0050	0.0201–0.0398	<0.001
Change, screw-retained restoration	0.0230	0.0033	0.0165–0.0295	<0.001
Difference in change, screw-retained vs. cement-retained	−0.0069	0.0060	−0.0187–0.0049	0.249

**Table 7:** Sensitivity of the estimated association between definitive retention and marginal bone level at the minimum 5-year follow-up across model specifications.

Model	B, mm	Robust SE	95% CI	*p*
Primary GEE without provisional retention	−0.0028	0.0070	−0.0165–0.0108	0.684
GEE including provisional retention	−0.0194	0.0097	−0.0385–−0.0004	0.045
GEE restricted to concordant retention strategies	−0.0021	0.0072	−0.0162–0.0119	0.766
Cluster-robust regression	−0.0027	0.0072	−0.0169–0.0115	0.707

## Data Availability

The de-identified data supporting the findings are available from the corresponding author on reasonable request, subject to ethical and institutional requirements.
